# Identification of a novel therapeutic target underlying atypical manifestation of Gaucher disease

**DOI:** 10.1002/ctm2.862

**Published:** 2022-05-20

**Authors:** Eun Na Kim, Hyo‐Sang Do, Hwangkyo Jeong, Taeho Kim, Sun Hee Heo, Yoo‐Mi Kim, Chong Kun Cheon, Yena Lee, Yunha Choi, In Hee Choi, Jeongmin Choi, Han‐Wook Yoo, Chong Jai Kim, Ari Zimran, Kyunggon Kim, Beom Hee Lee

**Affiliations:** ^1^ Computational Biology Program Department of Biomedical Engineering Oregon Health and Science University Portland Oregon USA; ^2^ Asan Institute for Life Sciences Asan Medical Center Seoul Republic of Korea; ^3^ Department of Biomedical Sciences University of Ulsan College of Medicine Seoul Republic of Korea; ^4^ AniCom Therapeutics Seoul Republic of Korea; ^5^ Department of Pediatrics Chungnam National University Sejong Hospital Sejong Republic of Korea; ^6^ Department of Pediatrics Pusan National University School of Medicine Research Institute for Convergence of Biomedical Science and Technology Pusan National University Yangsan Hospital Yangsan Republic of Korea; ^7^ Department of Pediatrics Asan Medical Center Children's Hospital University of Ulsan College of Medicine Seoul Republic of Korea; ^8^ Department of Genetic Counseling University of Ulsan College of Medicine Seoul Republic of Korea; ^9^ Department of Pathology Asan Medical Center University of Ulsan College of Medicine Seoul Republic of Korea; ^10^ The Hebrew University Hadassah Medical School Jerusalem Israel; ^11^ Department of Convergence Medicine Asan Medical Center University of Ulsan College of Medicine Seoul Republic of Korea; ^12^ Medical Genetics Center Asan Medical Center University of Ulsan College of Medicine Seoul Republic of Korea

Dear Editor,

In the present study, we delineate the molecular pathways underlying atypical progressions of Gaucher disease (GD) that lead to unresponsiveness to enzyme replacement therapy (ERT). Specifically, we observed the accumulation of dense substrates (e.g., glucosylsphingosine [Lyso‐Gb1]), which was associated with alterations in complement activity, autophagy metabolism, macrophage polarization and TGF‐β signaling and subsequent endothelial‐to‐mesenchymal transition (EndMT) and fibrosis. We also describe the potential therapeutic role of ambroxol, a chemical chaperone in GD, and highlight the need for a multi‐functional therapeutic approach in managing GD cases with atypical progression.

GD is caused by the deficiency of glucocerebrosidase (GCase) encoded by the glucocerebrosidase1 gene, which leads to the accumulation of glucosylceramide and Lyso‐Gb1 in the lysosome. Specifically, Lyso‐Gb1 plays important pathogenic roles in GD such as inflammation, impairment of cytoplasmic division, alteration of immune regulation and neurotoxicity.^1^, ^2^, ^3^ GD can be categorized according to the degree of neurologic involvement: non‐neuropathic (GD1), acute neuronopathic (GD2) and chronic neuronopathic (GD3). Since the advent of ERT, the clinical outcomes of patients with GD have improved remarkably; however, despite ERT, most GD2/3 patients as well as some GD1 patients show atypical manifestations such as lymphadenopathy, multiple myeloma, lymphoma, neurological deterioration and increases in Lyso‐Gb1 levels.^4^ The molecular mechanism underlying atypical responses to ERT is poorly understood.

We evaluated the histological features of lymph nodes from three patients with GD, including submandibular lymph node from Pt1 (GD1, G85E/F252I), stomach lymph node from Pt2 (GD1, R87W/R296Q) and mesenteric lymph node from Pt3 (GD3, L483P/L483P) (Table S1). Despite 5 years of ERT, Pt3 died from progressive mesenteric lymphadenopathy with protein‐losing enteropathy,^5^ and the mesenteric lymph nodes of Pt3 had Gaucher‐like cells with atypical histomorphology (Figure 1A and S1, red box) including larger and hyperchromatic nuclei with frequent multinucleations, abundant fibrosis and thick fibrous capsules (Figure 1B). The proinflammatory cytokine, MCP‐1, was expressed more intensely in atypical tissues (Pt3_GD3_AT) than in typical tissues from the same patient (Pt3_GD3_T); while the M1 and M2 marker CD68 was also strongly expressed in both areas, MRC1 and mannose receptors, representing M2 macrophages, were slightly over‐expressed in atypical tissues (Figure 1C). Immunointensities of C5a, C5a receptors, C1q and C4b were stronger in atypical tissues (Figure 1D), indicating a more intense activation of the classical complement pathway. Altered complement activity affects macrophage polarization by the intracellular accumulation of immune complexes and the secretion of proinflammatory cytokines from immune cells.^6^, ^7^

**FIGURE 1:**
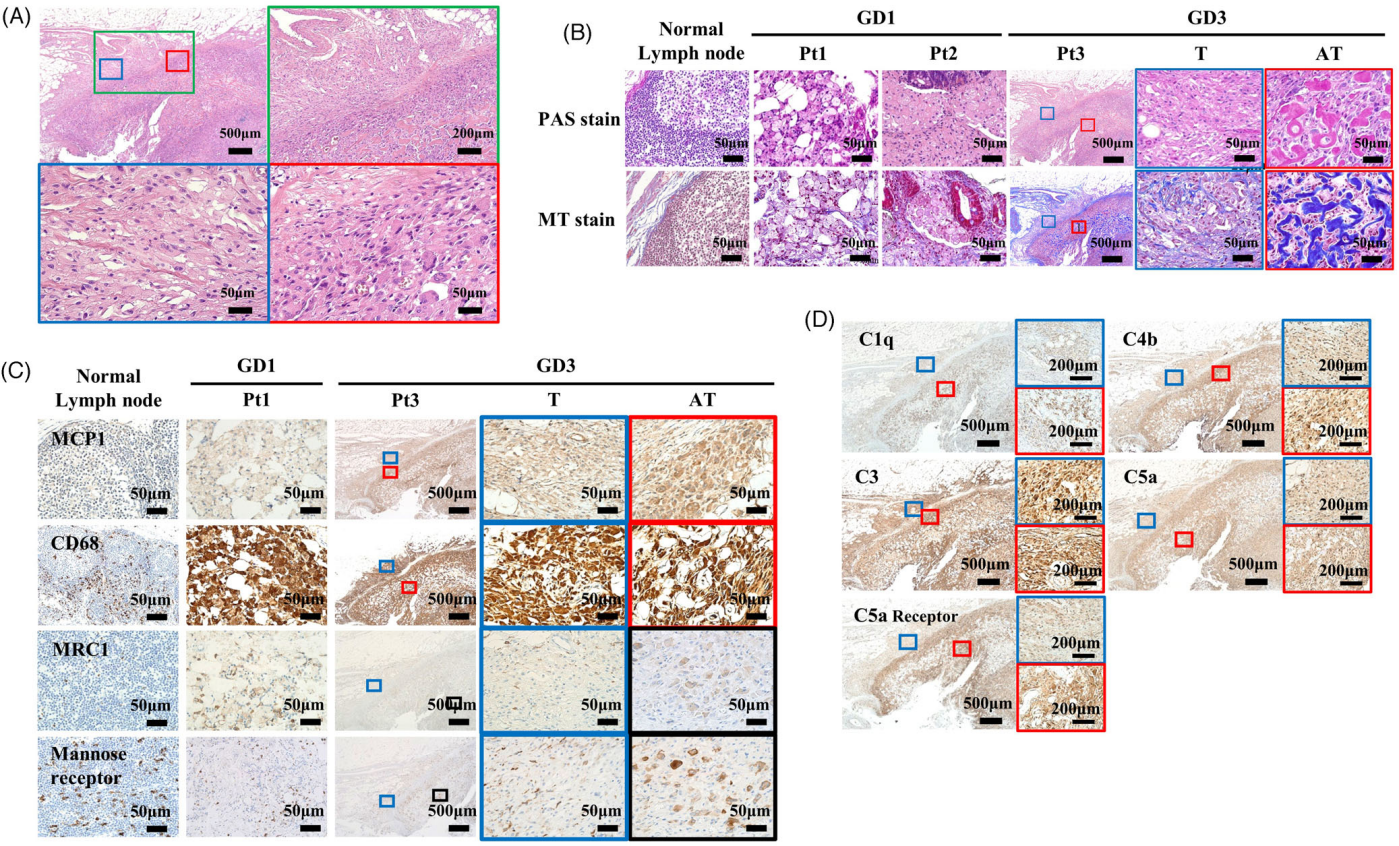
Histology and immunohistochemistry of the lymph nodes from Gaucher disease (GD) patients. (A) Haematoxylin and eosin staining of the lymph node from GD patients. Green, blue and red boxes show higher magnification views. Scale bars represent 500 μm (upper left panel), 200 μm (upper right panel; green box) and 50 μm (lower panel; blue and red boxes). (B) Periodic acid–Schiff (PAS) staining and Masson trichrome (MT) staining in Pt1_GD1_T, Pt3_GD3_T (blue box) and Pt3 _GD3_AT (red box). Blue and red boxes are higher magnification views of Pt3. Scale bars represent 50 μm and 500 μm. (C) Immunohistochemistry of macrophage markers (MCP‐1, CD68, MRC1 and Mannose receptor) in GD patients. Blue and red boxes are higher magnification views of Pt3. Scale bars represent 50 μm and 500 μm. (D) Immunohistochemistry of complement components (C5a, C5a receptors, C1q, C4b and C3). Blue and red boxes are higher magnification views. Scale bars represent 500 μm and 200 μm (blue and red boxes)

GSK‐3β, which inhibits mammalian Target of Rapamycin (mTOR) signaling, was increased in atypical tissues (Figure 2A). Atypical tissues also showed higher expressions of P62 (early endosome marker) and LC3AB (all‐stage endosome marker) (Figure 2B), indicating increased formations of autophagosomes. However, subsequent autophagy flux did not show a similar degree of increase as evidenced by the staining intensities of LAMP2A (lysosome marker), RAB7 (autolysosome, late‐endosome marker) and HSC70 (chaperone‐mediated autophagy marker) (Figure 2C). The conformation change from LC3 1/2 was not observed in the fibroblasts of Pt3 (Figure 2D). In addition, Pt3 fibroblasts showed a low expression of LAMP2, which is related to the lysosomal membrane, and a high expression of Beclin1, which modulates autophagy (Figure 2D).

**FIGURE 2:**
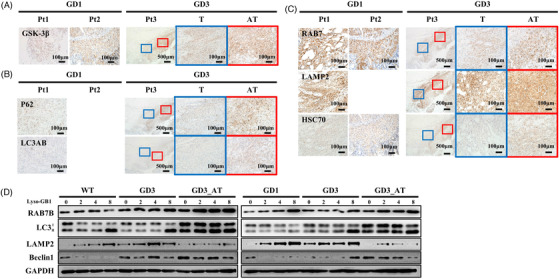
Expression of autophagy‐related proteins in Gaucher disease (GD) patients. (A) Immunohistochemistry of GSK‐3β in the lymph nodes of GD patients. Blue and red boxes are higher magnification views of Pt3. Scale bars represent 500 μm and 100 μm (blue and red boxes). (B) Immunohistochemistry of P62 and LC3AB. Blue and red boxes are higher magnification views of Pt3. Scale bars represent 500 μm and 100 μm. (C) Immunohistochemistry of RAB7, LAMP2 and HSC70. Blue and red boxes are higher magnification views of Pt3. Scale bars represent 500 μm and 100 μm. (D) Western blot analysis of autophagy‐related proteins in the fibroblasts of GD patients after treatment with the indicated concentrations (μM) of Lyso‐GB1. (GD1: D448H/L483P, GD3: P210fs*21/N227S, GD3_AT: p.L483P/L483P)

To assess the global proteomic profiles of the atyapical tissues of Pt3, proteomic analysis was performed using three samples—Pt1 (GD1), Pt3_GD3_T (typical) and Pt3_GD3_AT (atypical) (Figure S2). Principal component analysis revealed that the three samples had notably different characteristics (Figure 3A). We also found eight distinct clusters of proteins that showed differential expression in Pt3_GD3_AT compared with Pt1 and Pt3_GD3_T, including cluster_1 (580 proteins) and cluster_6 (156 proteins) (Figure 3B,C). In particular, the expression of the proteins differed in biological treatment, cellular component, molecular function and KEGG pathway (Figure 3D). Among these, MMP19, TIMP3, THBS2 and EGFL, which are associated with extracellular matrix (ECM) remodelling,^8^ were among the top 10 up‐regulated and top 10 down‐regulated proteins in atypical tissues (Table S2). Proteomics analysis showed that MMP, TIMP, EndMT and EMT were increased in atypical tissues (Figure S3). Importantly, Transforming Growth Factor‐β1 (TGF‐β1) and Thrombospondin‐1 (THBS1) were also highly over‐expressed in atypical tissues (Figure 3E), suggesting alterations in the EndMT process with ECM remodelling in Pt3_GD3_AT. Histological analysis showed that the expression levels of TGF‐β1 and TGF‐β receptors 1 and 2 were slightly higher in the atypical tissues (Figure 3F). Suppressor of Mothers Against Decapentaplegic 2 (SMAD2), p‐SMAD2, SMAD3, p‐SMAD3 and SMAD4 were strongly stained in atypical tissues (Figure 3G), as well as those of fibrosis markers Collagen 1 and Collagen 3 (Figure 3H). Notably, when patient‐derived fibroblasts were cultured and treated with Lyso‐Gb1, the levels of TGF‐β, ECM remodelling proteins (Collagens 1 and 3) and a fibrosis marker (SNAIL) were increased in GD3_AT but not in GD1 or GD3_T (Figure 3I). These findings suggest that an altered TGF‐ß signaling pathway, in association with altered complement^9^ and autophagy activities, plays an important role in the unresponsiveness to ERT in GD.

**FIGURE 3:**
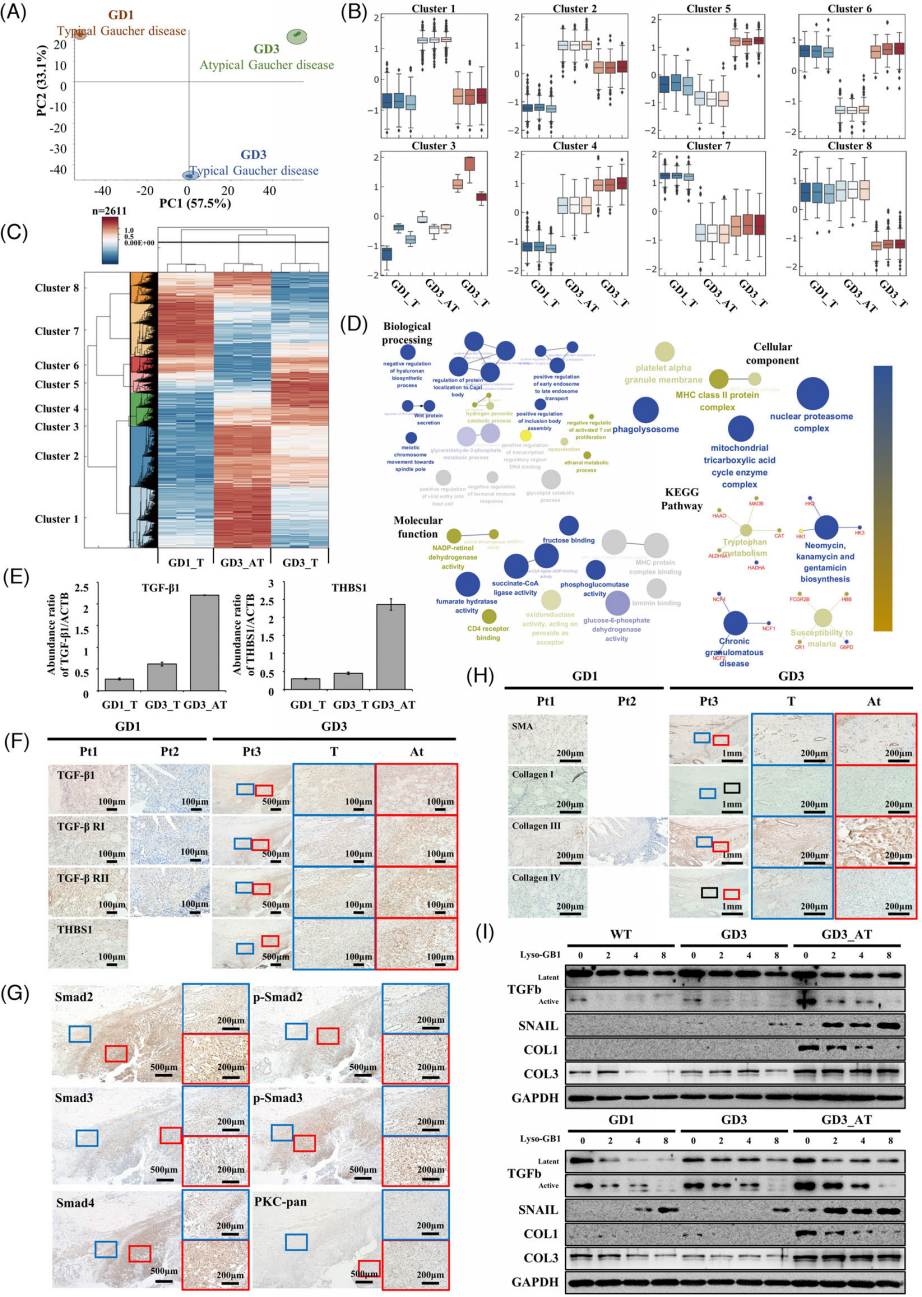
Proteomic analysis of Gaucher disease (GD) patients. (A) Principal components analysis of Pt1_GD1_T, Pt3_GD3_T and Pt3_GD3_AT. (B) Eight distinct protein clusters with different expression profiles in Pt1_GD1_T, Pt3_GD3_T and Pt 3 GD3_AT. (C) Heatmap and hierarchical clustering of the proteomic analysis data from Pt1_GD1_T, Pt3_GD3_T and Pt 3 GD3_AT. (D) Enrichment by gene ontology terms using Cytoscape plug‐in ClueGO between Pt3_GD3_T and Pt3_GD3_AT. (E) Increased expression of TGF‐β1 and THBS‐1 in Pt 3_GD3_AT compared with Pt1_GD1_T and Pt3_GD3_T. (F) Immunohistochemistry of TGF‐β1, TGF‐β receptor I, TGF‐β receptor II and THBS‐1. Blue and red boxes are higher magnification views of Pt3. Scale bars represent 500 μm and 100 μm (blue and red boxes). (G) Immunohistochemistry of SMAD signaling in Pt3_GD3. Blue and red boxes are higher magnification views. The scale bars represent 500 μm and 200 μm (blue and red boxes). (H) Immunohistochemistry of extracellular matrix (ECM)‐related proteins (Smooth Muscle Actin (SMA), Collagen I, Collagen III and Collagen IV). Blue and red boxes are higher magnification views of Pt3. Scale bars represent 1 mm and 200 μm (blue and red boxes). (I) Western blot analysis of fibroblasts from GD patients after treatment with the indicated concentrations (μM) of Lyso‐Gb1. (GD1: D448H/L483P, GD3: P210fs*21/N227S, GD3_AT: p.L483P/L483P)

Ambroxol acts as a chemical chaperone in certain glucocerebrosidase1 genotypes. As high‐dose ambroxol has a neuroprotective effect in GD3 and decreases Lyso‐Gb1,^10^ we investigated whether ambroxol carries additional roles in resolving the atypical progression in GD. High‐dose ambroxol treatment only slightly increased the GCase activity of fibroblasts from Pt3 with p.L483P homozygotes (Figure 4A) but reduced TGF‐β secretion in Wild Type (WT) fibroblasts treated with Lyso‐Gb1 (Figure 4B). Likewise, compared with that in untreated GD2/3 patients, the plasma level of TGF‐β was lower in GD3 patients who had been treated with high‐dose ambroxol for 2 or more years^10^ (Figure 4C). Moreover, ambroxol treatment down‐regulated TGF‐β receptor 1, Collagen 1 and Collagen 3 in Pt3 fibroblasts and up‐regulated LC3‐II in WT and GD3 fibroblasts (Figure 4D). Pt3 fibroblasts produced higher levels of p‐SMAD3, which was reduced by ambroxol treatment (Figure 4E). Ambroxol also reduced collagen deposition in Pt3 fibroblasts (Figure 4F). These findings collectively indicate that ambroxol exerts an anti‐inflammatory effect by stabilizing TGF‐β signaling and restoring autophagy metabolism in GD. However, a considerable number of ambroxol tablets would need to be consumed to achieve this high concentration, and the safe daily dosage of ambroxol in GD patients is unknown. Further research is required to develop a new agent with a more potent activity or one that modulates other therapeutic targets.

**FIGURE 4:**
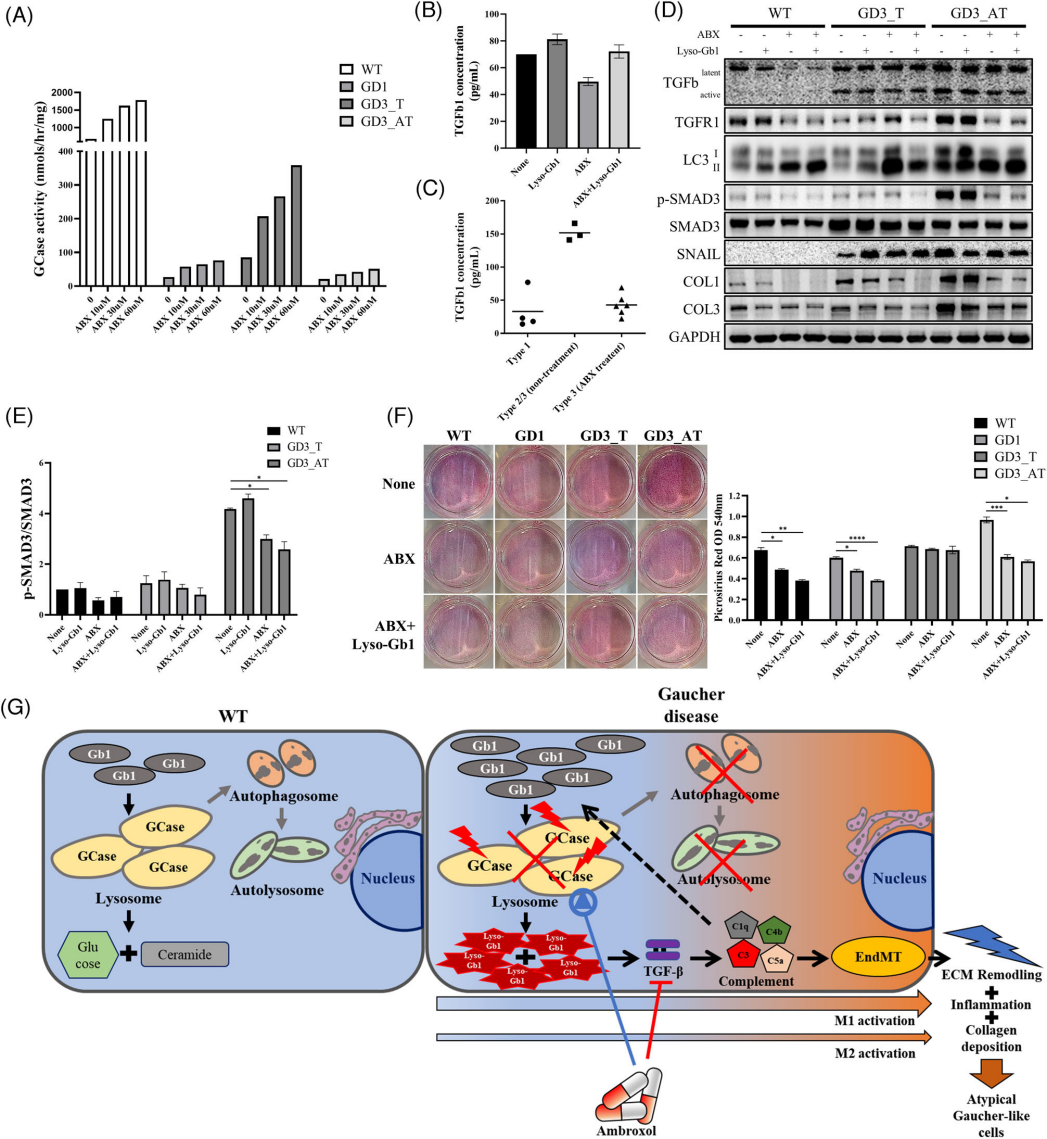
Effect of ambroxol (ABX) in the fibroblasts of Gaucher disease (GD) patients. (A) Gcase activity in GD fibroblasts treated with the indicated concentrations of ABX for 5 days. (B) TGF‐β1 secretion in WT fibroblasts treated with Lyso‐Gb1 (8μM) and ABX (60μM). Data are mean ± Standard Error of the Mean (SEM) (*n* = 2). (C) Plasma TGF‐β1 levels in GD patients. (D) Western blot analysis of GD fibroblasts after treatment with Lyso‐Gb1 and ABX. (GD3_T: P210fs*21/N227S, GD3_AT: p.L483P/L483P). (E) SMAD3 signaling in GD fibroblasts after treatment with Lyso‐Gb1 and ABX. Data are mean ± SEM (*n* = 3). *p < .05. (F) Collagen deposition in GD fibroblasts after treatment with Lyso‐Gb1 and ABX. Data are mean ± SEM (*n* = 3). *p < .05; **p < .01; ***p < .001; ****p < .0001. (G) Schematic model for lymphadenopathy in GD with atypical Gaucher tissue

In conclusion, we found that the atypical and devastating progression of GD is associated with dense Lyso‐Gb1 accumulation, aberrant autophagy metabolism, TGF‐β signaling and altered complement activity that collectively lead to EndMT and fibrosis (Figure 4G). In addition to ERT, pharmaceutical agents that can modulate these molecular alterations may be beneficial in disease management. Yet, as our histomorphological and proteomic studies were based on one patient (Pt3), these findings could have been drawn from his individual characteristics. Further studies in other patients with a similar phenotype to Pt3 are necessary.

## CONFLICT OF INTEREST

The authors declare that there is no conflict of interest that could be perceived as prejudicing the impartiality of the research reported.

## Supporting information

Supporting Information

## References

[1] Pandey MK , Jabre NA , Xu YH , Zhang W , Setchell KD , Grabowski GA . Gaucher disease: chemotactic factors and immunological cell invasion in a mouse model. Mol Genet Metab. 2014;111(2):163‐171. 10.1016/j.ymgme.2013.09.002. 24079945

[2] Pandey MK , Grabowski GA . Immunological cells and functions in Gaucher disease. Crit Rev Oncog. 2013;18(3):197‐220. 10.1615/critrevoncog.2013004503. 23510064 PMC3661296

[3] Revel‐Vilk S , Fuller M , Zimran A . Value of glucosylsphingosine (Lyso‐Gb1) as a biomarker in Gaucher disease: a systematic literature review. Int J Mol Sci. 2020;21(19):7159. 10.3390/ijms21197159. PMC7584006 32998334

[4] Elstein D , Mellgard B , Dinh Q , et al. Reductions in glucosylsphingosine (lyso‐Gb1) in treatment‐naïve and previously treated patients receiving velaglucerase alfa for type 1 Gaucher disease: data from phase 3 clinical trials. Mol Genet Metab. 2017;122(1‐2):113‐120. 10.1016/j.ymgme.2017.08.005. 28851512

[5] Lee JY , Lee BH , Kim GH , et al. Clinical and genetic characteristics of Gaucher disease according to phenotypic subgroups. Korean J Pediatr. 2012;55(2):48‐53. 10.3345/kjp.2012.55.2.48. 22375149 PMC3286762

[6] Pandey MK , Burrow TA , Rani R , et al. Complement drives glucosylceramide accumulation and tissue inflammation in Gaucher disease. Nature. 2017;543(7643):108‐112. 10.1038/nature21368. 28225753

[7] Serfecz JC , Saadin A , Santiago CP , et al. C5a activates a pro‐inflammatory gene expression profile in human Gaucher iPSC‐Derived macrophages. Int J Mol Sci. 2021;22(18):9912. 10.3390/ijms22189912. 34576075 PMC8466165

[8] Kessenbrock K , Plaks V , Werb Z . Matrix metalloproteinases: regulators of the tumor microenvironment. Cell. 2010;141(1):52‐67. 10.1016/j.cell.2010.03.015. 20371345 PMC2862057

[9] Gu H , Mickler EA , Cummings OW , et al. Crosstalk between TGF‐β1 and complement activation augments epithelial injury in pulmonary fibrosis. FASEB J. 2014;28(10):4223‐4234. 10.1096/fj.13-247650. 24958208 PMC4202097

[10] Kim YM , Yum MS , Heo SH , et al. Pharmacologic properties of high‐dose ambroxol in four patients with Gaucher disease and myoclonic epilepsy. J Med Genet. 2020;57(2):124‐131. 10.1136/jmedgenet-2019-106132. 31649052 PMC7029246

