# Supplementary material for: Identification of a novel therapeutic target underlying atypical manifestation of Gaucher disease
Source: Clin Transl Med. 2022 May 20;12(5):e862. doi: 10.1002/ctm2.862 (PMC9121313; doi:10.1002/ctm2.862)
Supplement: Supplementary file 1 — Supporting Information [file CTM2-12-e862-s001.docx]

**Supplementary Information**

**Methods**

**Formalin-Fixed Paraffin-Embedded (FFPE) tissue preparation**

FFPE tissue blocks were used for proteomic analysis. For the analysis of Pt3 with atypical GD3,a pathologist (E.N.K) meticulously dissected the H&E-stained slides of FFPE tissue blocks into areas with typical Gaucher cell morphology and those with atypical Gaucher cell morphology under direct microscopic visualization.

FFPE tissue sections were collected in a 1.5 mL tube, and 500 µL of heptane was added into the tube and incubated for 1 hour at room temperature for deparaffinization. After adding 25 uL of methanol, the pellet was collected using centrifugation at 9000 × g for 2 min. Extraction buffer EXB in Qproteome FFPE tissue kit (Qiagen) with 6 µL of *β*-mercapto-ethanol was added to the pellet. Then, the samples were incubated in a thermomixer at 100 °C for 20 min and then at 80°C for 2 h. The tube was placed on ice for 1 min and centrifuged for 15 min at 14,000 × g at 4 °C. The supernatant containing the extracted proteins was transferred to a new tube. After precipitation using cold acetone, the supernatant was removed, and the pellet was mixed with 100 µl of 8 M urea for protein quantification with a BCA assay.

**Enzymatic digestion with in-solution digestion method**

Sample preparation for FFPE proteome analysis was performed as described in our previous study.^1^ Briefly, samples of paraffin block materials were collected in 1.5 ml Eppendorf^®^ LoBind microcentrifuge tubes (Sigma-Aldrich). For deparaffinization and protein extraction, Qproteome FFPE tissue kit (Qiagen, Hilden, Germany) was used.

The extracted protein mixture from the FFPE tissue section was reduced using 5 mM Tris (2-carboxyethyl) phosphine for 1 hour at room temperature, followed by alkylation of cysteines with 15 mM iodoacetamide in 50 mM NH_4_HCO_3_ for 1 hour in the dark. Finally, each sample was treated overnight with 0.1 µg/µL of sequencing grade modified trypsin/Lys-C mixture (Promega) in 50 mM NH_4_HCO_3_ buffer at 37℃. The digestion reaction was stopped by adding 0.1% formic acid, and the resulting peptide mixture was desalted using Sep-Pak reverse-phase cartridges (Waters) and dried.

**Peptidization for mass spectrometry analysis**

Peptidization for mass spectrometry analysis was performed as described in our previous study.^1^ Briefly, The extracted proteins were reduced with 20 mM dithiothreitol and alkylated using iodoacetamide. Samples were loaded onto an S-Trap mini spin column (Protifi, NY, USA) and digested with trypsin/LysC. Samples were sequentially eluted with 50 mM triethylammonium bicarbonate, 0.2% formic acid, and 50% acetonitrile/0.2% formic acid. The combined eluates were dried using a speed vacuum and stored at -20°C.

**Statistical analysis for proteome data**

Statistical analysis for proteome data was performed as described in our previous analysis.^1^ The Perseus software (version 1.6.8.0)^2^ was used for statistical analysis of the relative abundance of the proteins among the samples. The values of the normalized protein abundances were transformed into the log_2_ scale. Three technical replicates of each sample were grouped, and proteins that had at least three frequencies among three sample sets were taken as the valid value. Missing value imputation of the peptides was performed from a normal distribution. Student’s *t*-test was performed using permutation-based FDR (cut-off, 0.01) for volcano plots. Hierarchical clustering was performed after z-score normalization. Gene ontology analyses were performed using web-based tools, including the g: Profiler (<https://biit.cs.ut.ee/gprofiler/gost>) and the Enrichr (https://amp.pharm.mssm.edu/Enrichr/).

**Ingenuity pathway analysis of differentially expressed proteins**

Ingenuity pathway analysis (IPA) of differentially expressed proteins (DEPs) was performed as described in our previous analysis.^1^ The biological functions and enriched canonical pathways were based on Z-scores and fold changes. Significantly enriched pathways were recognized using a cut-off of *P* < 0.01. The molecule activity predictor was used to predict the upstream activation or inhibition of a pathway.

**Western blotting**

For isolation of total proteins, cells were lysed in PRO-PREM^TM^ (Intron Biotechnology). The protein concentration of the supernatant was quantified by Bradford protein assay (Bio-Rad Hercules, CA, USA). Total protein (20 µg) was loaded on SDS-PAGE and then transferred to a nitrocellulose membrane (Bio-Rad). The membranes were blocked for 1 h with 4% skim milk in Tris-Buffed saline (TBS) containing 0.1% Tween 20. The membranes were incubated overnight at 4°C with the primary antibodies and then washed with TBST. The membranes were incubated for 1 h with HRP-conjugated secondary antibody in blocking solution at room temperature. After washing with TBST, specific bands on the membrane were analyzed using Ez-Capture MG (ATTO CORPORATION, Tokyo, Japan) according to the manufacturer’s protocol.

**Supplementary Table 1. Clinical and genetic characteristics of three patients with Gaucher disease**

| **Patient** | | **Pt1** | **Pt2** | **Pt3** |
| --- | --- | --- | --- | --- |
| Sex/Age at diagnosis | | M/12yr | M/35y | M/4yr |
| Type | | GD1 | GD1 | GD3 |
| GBA mutation | Genotype 1 | c.254G>A (p.Gly85Glu) | c.259C>T (p.Arg87Trp) | c.1448T>C (p.Leu483Pro) |
|  | Genotype 2 | c.754T> A (p.Phe252Ile) | c.887G>A (p.Arg296Gln) | c.1448T>C (p.Leu483Pro) |
| Hepatomegaly | | YES | YES | YES |
| Splenomegaly | | YES | YES | YES |
| Neurological Manifestation | | None | None | Intellectual disability, strabismus |
| Other findings | | Avascular necrosis of femur neck | Avascular necrosis of femur neck | Mesenteric lymphadenopathy, kyphosis, stunting growth |
| Biopsy tissue | | Mandibular lymph node | Duodenal mucosa | Mesenteric lymph nodes |

**Supplementary Table 2.** **List of top 10 and bottom 10 proteins with differential expression between atypical Gaucher cell tissues and typical Gaucher cell tissues in a GD3 patient**

|  | **Accession** | **Description** | **Gene** | **Log_2_ (Atypical/Typical)** | **-Log_10_ (P value)** |
| --- | --- | --- | --- | --- | --- |
| **High** | Q99542 | Matrix metalloproteinase-19 | MMP19 | 9.29421 | 7.5517 |
|  | P35442 | Thrombospondin-2 | THBS2 | 8.29015 | 3.37356 |
|  | Q86UD1 | Out at first protein homolog | OAF | 7.29531 | 5.44336 |
|  | P35625 | Metalloproteinase inhibitor 3 | TIMP3 | 7.17457 | 7.91382 |
|  | Q641Q3 | Meteorin-like protein | METRNL | 7.07962 | 3.03533 |
|  | Q8NES3 | Beta-1,3-N-acetylglucosaminyltransferase lunatic fringe | LFNG | 7.01573 | 9.672 |
|  | Q9BUT1 | 3-hydroxybutyrate dehydrogenase type 2 | BDH2 | 6.99024 | 6.60889 |
|  | P26927 | Hepatocyte growth factor-like protein | MST1 | 6.67985 | 8.21098 |
|  | Q92743 | Serine protease HTRA1 | HTRA1 | 6.42661 | 4.9717 |
|  | O75487 | Glypican-4 | GPC4 | 6.26037 | 5.31324 |
| **Low** | Q9UHF1 | Epidermal growth factor-like protein 7 | EGFL7 | -7.73294 | 5.62405 |
|  | Q9UEY8 | Gamma-adducin | ADD3 | -7.52608 | 6.80763 |
|  | P55854-2 | Isoform 2 of Small ubiquitin-related modifier 3 | SUMO3 | -7.21534 | 3.422 |
|  | P00750 | Tissue-type plasminogen activator | PLAT | -5.93074 | 4.46086 |
|  | P02686 | Myelin basic protein | MBP | -5.30773 | 3.43999 |
|  | Q13595 | Transformer-2 protein homolog alpha | TRA2A | -5.14961 | 3.5235 |
|  | Q9UH65 | Switch-associated protein 70 | SWAP70 | -5.03759 | 2.9319 |
|  | Q8WU39 | Marginal zone B- and B1-cell-specific protein | MZB1 | -4.9036 | 4.46646 |
|  | Q5VZ66 | Janus kinase and microtubule-interacting protein 3 | JAKMIP3 | -4.86221 | 5.20634 |
|  | Q5HYK3-2 | Isoform 2 of 2-methoxy-6-polyprenyl-1,4-benzoquinol methylase, mitochondrial | COQ5 | -4.83695 | 3.23342 |

**Supplementary Table 3. Antibodies for immunohistochemistry**

| No | Antibody | Host | Isotype | Dilution | Cat. No. | Company |
| --- | --- | --- | --- | --- | --- | --- |
| 1 | Anti-GLCSPH | Rabbit | Polyclonal IgG | 1:50 | #111584 | Antibody Research |
| 2 | Anti-C1q | Rabbit | Monoclonal IgG | 1:200 | ab75756 | Abcam |
| 3 | Anti-C4b | Rabbit | Polyclonal IgG | 1:50 | ab66791 | Abcam |
| 4 | Anti-C3a | Mouse | Monoclonal IgG | 1:200 | LS-b15388 | LSbio |
| 5 | Anti-C5a | Rabbit | Polyclonal IgG | 1:200 | ab193295 | Abcam |
| 6 | Anti-C5a receptor | Rabbit | Polyclonal IgG | 1 µg/ml | ab59390 | Abcam |
| 7 | Anti-CD68 | Mouse | Monoclonal IgG | 1:200 | M0814 | DAKO |
| 8 | Anti-Mannose receptor | Rabbit | Polyclonal IgG | 0.1 µg/ml | ab64693 | Abcam |
| 9 | Anti-CD163 | Mouse | Monoclonal IgG | 1:400 | 163M-16 | CELL MARQUE |
| 10 | Anti-MRC1 | Mouse | Monoclonal IgG1 | 1.5 µg /ml | H00004360-M02 | Abnova |
| 11 | Anti-pGSK-3b | Rabbit | Monoclonal IgG | 1:50 | #9323 | Cell Signaling |
| 12 | Anti-GSK-3b | Rabbit | Monoclonal IgG | 1:100 | #9315 | Cell Signaling |
| 13 | Anti- S6K1 | Rabbit | Monoclonal IgG | 1:10000 | #2708 | Cell Signaling |
| 14 | Anti-P62 | Mouse | Monoclonal IgG1 | 1:1000 | #88588 | Cell Signaling |
| 15 | Anti-LC3AB | Rabbit | Monoclonal IgG | 1:500 | #12741 | Cell Signaling |
| 17 | Anti-RAB7 | Rabbit | Monoclonal IgG | 1:100 | ab137029 | Abcam |
| 18 | Anti-LAMP2 | Rabbit | Polyclonal IgG | 1:500 | ab18528 | Abcam |
| 19 | Anti-HSC70 | Rabbit | Monoclonal IgG | 1:50 | ab51052 | Abcam |
| 20 | Anti-MCP-1 | Rabbit | Polyclonal IgG | 1:35 | Ab9669 | Abcam |
| 21 | Anti-TGF-β1 | Mouse | Monoclonal IgG1 | 1:50 | NBP2-45137 | Novus Biologicals |
| 22 | Anti-TGF-β receptor 1 | Rabbit | Polyclonal IgG | 1:50 | ab31013 | Abcam |
| 23 | Anti-TGF-β receptor 2 | Mouse | Monoclonal IgG1 | 1:50 | ab78419 | Abcam |
| 24 | Anti-THBS1 | Mouse | Monoclonal IgG1 | 1:100 | ab1823 | Abcam |
| 25 | Anti-Smad2 | Rabbit | Polyclonal IgG | 1:100 | ab63576 | Abcam |
| 26 | Anti-p-Smad2 | Rabbit | Polyclonal IgG | 1:100 | 44-244G | Invitrogen |
| 27 | Anti-Smad3 | Rabbit | Monoclonal IgG | 1:500 | ab40854 | Abcam |
| 28 | Anti-p-Smad3 | Rabbit | Monoclonal IgG | 1:100 | ab52903 | Abcam |
| 29 | Anti-Smad4 | Rabbit | Monoclonal IgG | 1:1000 | 1676-1 | EPITOMICS |
| 30 | Anti-a-SMA | Mouse | Monoclonal IgG |  | M0851 | DAKO |
| 31 | Anti-Collagen I | Rabbit | Polyclonal IgG | 1:400 | ab34710 | Abcam |
| 32 | Anti-Collagen III | Rabbit | Polyclonal IgG | 1:1000 | ab7778 | Abcam |
| 33 | Anti-Collagen IV | Rabbit | Polyclonal IgG | 1:2000 | ab6586 | Abcam |
| 34 | Anti-MHC class I | Mouse | Monoclonal IgG | 1:10000 | 2708 | Cell Signaling |
| 35 | Anti-KI-67 | Mouse | Monoclonal IgG | 1:200 | M7240 | DAKO |
| 36 | Anti-PKC | Rabbit | Polyclonal IgG | 1:200 | PA5-36757 | Invitrogen |

**Supplementary Figures**

**Supplementary Figure 1**. (A) Atypical Gaucher-like cells with multinucleated cells morphology in Pt3 GD3. Neck lymph node, mesenteric lymph node, mesenteric mass, and small bowel lymph nodes are collected from 2009 to 2014.


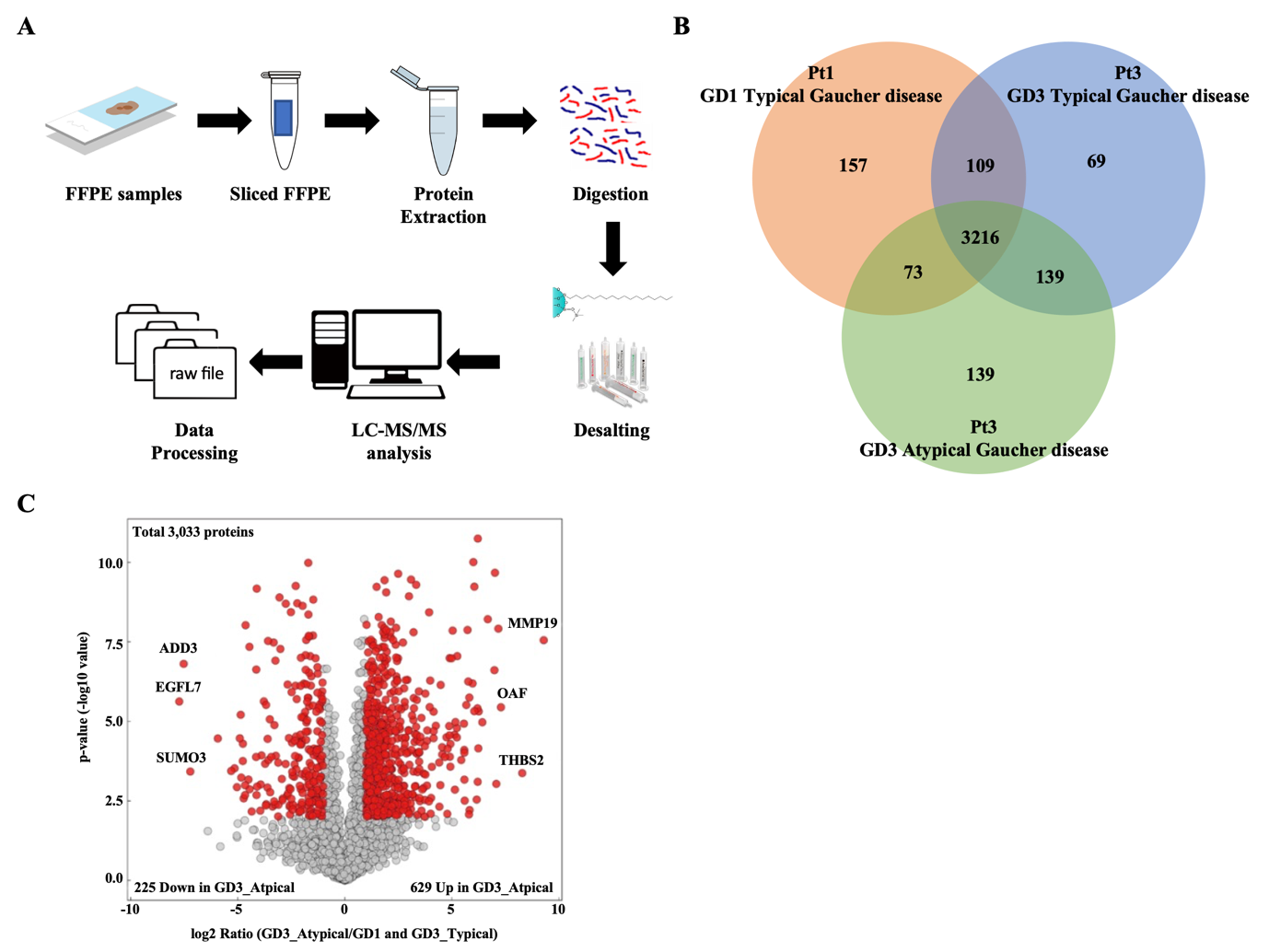


**Supplementary Figure 2**. (A) Schematic protocol of proteomic analysis. (B) Venn diagram of differentially expressed proteins from Pt1_GD1, Pt3_GD3_T, and Pt3_GD3_AT. (C) Volcano plot of differentially expressed proteins. Each dot corresponds to a potentially differentially expressed protein.

**3**

333

3

3

**Reference**

1. Kim EN, Yu J, Lim JS, et al. CRP immunodeposition and proteomic analysis in abdominal aortic aneurysm. *PLOS ONE*. 2021;16(8):e0245361. doi:10.1371/journal.pone.0245361

2. Tyanova S, Cox J. Perseus: A Bioinformatics Platform for Integrative Analysis of Proteomics Data in Cancer Research. In: von Stechow L, ed. *Cancer Systems Biology: Methods and Protocols*. Springer New York; 2018:133-148.

3. Kessenbrock K, Plaks V, Werb Z. Matrix metalloproteinases: regulators of the tumor microenvironment. *Cell*. Apr 2 2010;141(1):52-67. doi:10.1016/j.cell.2010.03.015

4. Ciszewski WM, Sobierajska K, Wawro ME, et al. The ILK-MMP9-MRTF axis is crucial for EndMT differentiation of endothelial cells in a tumor microenvironment. *Biochimica et Biophysica Acta (BBA) - Molecular Cell Research*. 2017/12/01/ 2017;1864(12):2283-2296. doi:<https://doi.org/10.1016/j.bbamcr.2017.09.004>

5. Piera-Velazquez S, Li Z, Jimenez SA. Role of endothelial-mesenchymal transition (EndoMT) in the pathogenesis of fibrotic disorders. *Am J Pathol*. 2011;179(3):1074-1080. doi:10.1016/j.ajpath.2011.06.001

6. Zhou BP, Deng J, Xia W, et al. Dual regulation of Snail by GSK-3beta-mediated phosphorylation in control of epithelial-mesenchymal transition. *Nat Cell Biol*. Oct 2004;6(10):931-40. doi:10.1038/ncb1173
